# Retraction Note: Dietary patterns and their associations with socio-demographic and lifestyle factors in Tasmanian older adults: a longitudinal cohort study

**DOI:** 10.1038/s41430-019-0541-7

**Published:** 2019-12-05

**Authors:** Hoa H. Nguyen, Feitong Wu, Wendy H. Oddy, Karen Wills, Sharon L. Brennan-Olsen, Graeme Jones, Tania Winzenberg

**Affiliations:** 1 Menzies Institute for Medical Research, University of Tasmania, 17 Liverpool Street, Hobart, TAS 7000 Australia; 2 University of Medicine and Pharmacy, 217 Hong Bang Street, District 5, Ho Chi Minh City, Vietnam; 3 Department of Medicine-Western Health, The University of Melbourne, 176 Furlong Road, St Albans, VIC 3021 Australia; 4 Australian Institute for Musculoskeletal Science (AIMSS), 176 Furlong Road, St Albans, VIC 3021 Australia; 5 Faculty of Health, University of Tasmania, 17 Liverpool Street, Hobart, TAS 7000 Australia

**Retraction Note to: European Journal of Clinical Nutrition** (2019) 73:714–723

10.1038/s41430-018-0264-1

The authors have retracted this article [1] after discovering a major error in the data analysis made when generating the grouped items used in the factor analysis generating the dietary patterns. Because the error is in the foundations of the analysis, it means that the dietary patterns identified were themselves erroneous and their associations with socio-demographic factors are also incorrect. A re-analysis showed up major differences in outcomes when compared with those in [1]. The authors have been given the opportunity to submit a new manuscript for peer review. All authors agree with this retraction.

[1] Hoa H. Nguyen, Feitong Wu, Wendy H. Oddy, Karen Wills, Sharon L. Brennan-Olsen, Graeme Jones & Tania Winzenberg. Dietary patterns and their associations with socio-demographic and lifestyle factors in Tasmanian older adults: a longitudinal cohort study. 2019;73:714–723.

